# Free surfaces recast superconductivity in few-monolayer MgB_2_: Combined first-principles and ARPES demonstration

**DOI:** 10.1038/s41598-017-13913-z

**Published:** 2017-10-31

**Authors:** J. Bekaert, L. Bignardi, A. Aperis, P. van Abswoude, C. Mattevi, S. Gorovikov, L. Petaccia, A. Goldoni, B. Partoens, P. M. Oppeneer, F. M. Peeters, M. V. Milošević, P. Rudolf, C. Cepek

**Affiliations:** 1 Department of Physics, University of Antwerp, Groenenborgerlaan 171, B-2020 Antwerp, Belgium; 2 Zernike Institute for Advanced Materials, University of Groningen, Nijenborgh 4, 9747AG Groningen, The Netherlands; 3 Present Address: Elettra Sincrotrone Trieste, Strada Statale 14 km.163.5, I-34149 Trieste, Italy; 4 Department of Physics and Astronomy, Uppsala University, Box 516, SE-751 20 Uppsala, Sweden; 5 IOM-CNR, Laboratorio TASC, Strada Statale 14 km.163.5, I-34149 Trieste, Italy; 6 Present Address: Department of Materials, Imperial College London, Exhibition road, SW7 2AZ London, United Kingdom; 7 Elettra Sincrotrone Trieste, Strada Statale 14 km.163.5, I-34149 Trieste, Italy; 8 Present Address: Canadian Light Source Inc., 44 Innovation Blvd, Saskatoon, SK S7N 2V3 Canada

## Abstract

Two-dimensional materials are known to harbour properties very different from those of their bulk counterparts. Recent years have seen the rise of atomically thin superconductors, with a caveat that superconductivity is strongly depleted unless enhanced by specific substrates, intercalants or adatoms. Surprisingly, the role in superconductivity of electronic states originating from simple free surfaces of two-dimensional materials has remained elusive to date. Here, based on first-principles calculations, anisotropic Eliashberg theory, and angle-resolved photoemission spectroscopy (ARPES), we show that surface states in few-monolayer MgB_2_ make a major contribution to the superconducting gap spectrum and density of states, clearly distinct from the widely known, bulk-like *σ*- and *π*-gaps. As a proof of principle, we predict and measure the gap opening on the magnesium-based surface band up to a critical temperature as high as ~30 K for merely six monolayers thick MgB_2_. These findings establish free surfaces as an unavoidable ingredient in understanding and further tailoring of superconductivity in atomically thin materials.

## Introduction

The number of materials superconducting down to monolayer thickness is rapidly growing over recent years^1,2^, now ranging from elemental metals, such as In and Pb grown on Si(111)^3–11^ and Ga^12^, to compound materials. The latter category comprises both superconductors with conventional electron-phonon interaction as the coupling mechanism, for instance transition metal dichalcogenides like NbSe_2_^13–16^, graphene doped with Li^17–19^, Ca^20,21^ and Ba^22^, Mo_2_C^23^, etc., and ones with non-conventional coupling mechanisms, for example La_2−*x*_Sr_*x*_CuO_4_ on insulating La_2_CuO_4_^24^ and FeSe on a SrTiO_3_ substrate^25–29^. Other new ultrathin superconductors have been predicted in silico recently, e.g., metal-intercalated blue phosphorus^30^. The realization of atomically thin superconductors fosters exciting prospects for materials engineering on the atomistic scale. Notably, they are ideally suited as building blocks for ultrathin and ultra-lightweight cryogenic electronic circuits^31–33^ where their quasi two-dimensional structure even allows more complex architectures to be constructed as, e.g., van der Waals heterostructures^34^. A limiting factor for these applications is the need for specific intercalants, adatoms or substrates in most current realizations of two-dimensional superconductivity. Some materials even need to be gated in order to establish superconductivity, e.g., two-dimensional MoS_2_^35^. Surprisingly, the exploitation of the electronic states of the free surfaces of two-dimensional superconductors has remained an almost completely unexplored terrain.

A particularly interesting candidate to examine the effect of thinning on superconductivity is hexagonal magnesium diboride (MgB_2_), owing to its conveniently layered, graphene-related structure as well as to its high critical temperature of 39 K in bulk form. MgB_2_ consists of alternating Mg- and B-planes, with stronger in-plane than out-of-plane electronic bonds. As a result, the electronic structure and superconducting properties of bulk MgB_2_ are strongly anisotropic, instigating the formation of two superconducting gaps, each on a different part of the Fermi surface^36–41^. A first-principles approach to the Eliashberg formalism has shown that the stronger gap, ${{\rm{\Delta }}}_{\sigma }\mathrm{(0)}\sim 7$ meV, in bulk MgB_2_ stems from the in-plane *σ*-bond, while the weaker gap, ${{\rm{\Delta }}}_{\pi }\mathrm{(0)}\sim 2-3$ meV, is connected to the out-of-plane *π*-bonds^40–42^. Superconductivity is fully governed by B-atoms in bulk MgB_2_^36–38,41^, since both the *σ*- and *π*-gaps originate from electrons in B-planes that couple to lattice vibrations of the B-atoms. MgB_2_ and other multigap superconductors are known to harbour rich new physics^43–47^, as a result of coupling, competition and phase frustration between different band condensates. Owing to the fact that *σ*-bands lie in-plane and *π*-bands out-of-plane, the layered, anisotropic crystal structure gives rise to innate multigap superconductivity in MgB_2_, while also enabling growing the material at atomic thickness, layer by layer. It is therefore of particular interest to understand how superconductivity would change in the thinnest limit. Hints of additional superconducting gaps opening in 2D materials have been suggested in recent works, for materials such as two-dimensional Ga and FeSe^48^. They are deduced from fitting of the measured temperature dependence of, e.g., the London penetration depth, which unfortunately does not provide much insight into the origin of the proposed additional gaps. The fitting technique is moreover not fully waterproof, as the measurements can equally result from just anisotropy of the superconducting gap, and no additional gap opening, as shown and discussed in ref.^47^.

Here, we discuss the nucleation of multigap superconductivity in atomically thin MgB_2_, and the crucial role of the electronic states of the free surface therein. The latter remained completely unexplored to date, as the previous studies mentioned above did not consider the role of surface states originating from free surfaces. Our analysis starts from a first-principles study of the structural and electronic properties of monolayer to few-monolayer MgB_2_, that exposes the origin of the emerging surface state. Secondly, we realized experimentally one to eight monolayers (MLs) of MgB_2_ using molecular beam epitaxy, and using *in situ* room- and low-temperature angle-resolved photoemission spectroscopy (ARPES) we validate the theoretical predictions for the surface state. Finally, by comparing *ab initio* anisotropic Eliashberg theory results and the measured superconducting gap for six MLs MgB_2_, we reveal the surprisingly distinct and influential contribution of the surface states to the superconducting gap spectrum and tunneling properties of few-monolayer MgB_2_. We show furthermore that the emerging superconductivity in the surface state interacts with the contributions due to *σ*- and *π*-bands, leading to novel multigap phenomena in ultrathin MgB_2_, different for each additional monolayer.

## Results

### Crystal structure

We begin with structural characterizations. Bulk MgB_2_ consists of hexagonal Mg layers intercalated with B layers in a honeycomb lattice. It thus adopts the hexagonal space group P6/mmm (No. 191)^49^. Mg occupies Wyckoff position $1a$, i.e., $\mathrm{(0,0,0)}$, while B occupies Wyckoff position $2d$, i.e., $(\frac{1}{3},\frac{2}{3},\frac{1}{2})$ and $(\frac{2}{3},\frac{1}{3},\frac{1}{2})$. We studied the crystal structure of few-monolayer MgB_2_ by performing a structural relaxation within density functional theory (DFT), as implemented in VASP^50^. We found no tendency to break the in-plane symmetry (no buckling, etc.) in one to eight monolayers (MLs) of MgB_2_, neither in freestanding form, nor with a Mg-substrate.

**Figure 1:**
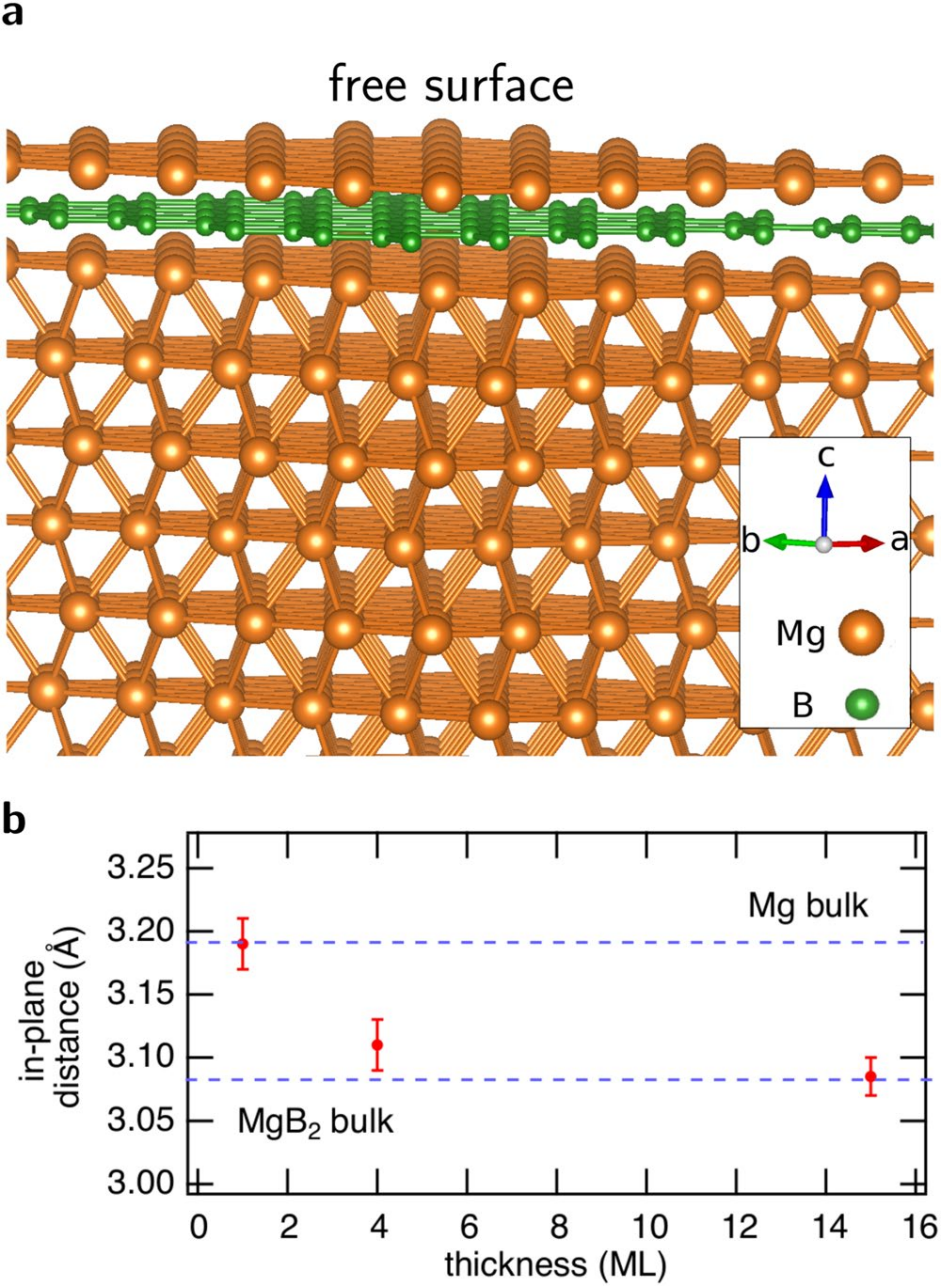
Crystal structure of few-monolayer MgB_2_. (**a**) Atomistic picture of a single Mg-terminated monolayer MgB_2_ on a Mg(0001) substrate. The inset shows the crystal axes and the atomic species. (**b**) The evolution with thickness of the in-plane lattice parameter of few-monolayer MgB_2_, measured using surface X-ray diffraction (SXRD).

The crystal structure of one ML of MgB_2_ on a Mg-substrate is depicted in Fig. 1a. The calculated in-plane lattice parameter of the Mg substrate is slightly larger than that of a freestanding ML of MgB_2_ (3.16 Å compared with 3.09 Å), resulting in an advantageously small lattice mismatch (below $3$%) in case the ML is grown on this substrate. The calculated interlayer distance evolves from 3.34 Å for one ML to 3.49 Å for eight MLs, steadily converging towards the bulk value, 3.52 Å. Few-monolayer MgB_2_ on a substrate can adopt two different forms, namely with B- or Mg-termination. To find out which termination is energetically favoured, we calculate the heat of formation of both possible structures. The heat of formation per atom, ${H}_{{\rm{f}}}({{\rm{Mg}}}_{x}{{\rm{B}}}_{y})$, of the binary structures with stoichiometry Mg_*x*_B_*y*_, including the Mg-substrate, is calculated as follows:

1 $$\begin{array}{cc}{H}_{{\rm{f}}}({{\rm{M}}{\rm{g}}}_{x}{{\rm{B}}}_{y}) & =\,\frac{1}{x+y}[{E}_{{\rm{t}}{\rm{o}}{\rm{t}}}({{\rm{M}}{\rm{g}}}_{x}{{\rm{B}}}_{y})-x\frac{{E}_{{\rm{t}}{\rm{o}}{\rm{t}}}({\rm{e}}{\rm{l}}{\rm{e}}{\rm{m}}.{{\rm{M}}{\rm{g}}}_{2})}{2}-y\frac{{E}_{{\rm{t}}{\rm{o}}{\rm{t}}}({\rm{e}}{\rm{l}}{\rm{e}}{\rm{m}}.{{\rm{B}}}_{12})}{12}].\end{array}$$

Here, the total energies are calculated using DFT. The total energy of elemental metallic ${{\rm{Mg}}}_{2}$ was calculated in its hexagonal form (identical to the substrate), while for ${{\rm{B}}}_{12}$ the trigonal $\alpha$-phase was selected. The results are listed in Table 1. Mg-terminated structures consistently have a lower heat of formation compared with B-terminated structures for more than one ML, the difference being in the order of a few tens of meV per atom. For one ML, the difference in heat of formation of both terminations is small. We therefore find that Mg-termination is preferred in few-monolayer MgB_2_. The Mg-atoms at the free surface stand in contrast with the B-dominated fermiology of bulk MgB_2_. This is the physical origin of all findings that will be discussed further in this article.

**Table 1:** Heat of formation, ${H}_{{\rm{f}}}$, in units of eV/atom, as a function of the number of MLs for B- and Mg-termination, as well as the difference in heat of formation between B- and Mg-termination.

Number of MLs	B-terminated	Mg-terminated	Difference
1	0.1211	0.1260	−0.0049
2	0.0761	−0.0114	0.0875
4	0.0030	−0.0542	0.0572
6	−0.0354	−0.0781	0.0427
8	−0.0593	−0.0935	0.0342

On the experimental side, we grew MgB_2_ films on a Mg(0001) substrate using molecular beam epitaxy, using a technique we developed for high-purity and high-quality films^51,52^. The Mg(0001) substrate was selected because of the minimal lattice mismatch with few-monolayer MgB_2_ as mentioned above. The thickness of the samples was monitored *in situ* by a combination of photoemission and calibrated evaporators, a technique explained in more detail in the Methods section, and in refs^51,52^. To characterize the lattice parameters of the experimental samples, we carried out surface X-ray diffraction (SXRD) experiments. The results are depicted in Fig. 1b. For the thinnest films, the in-plane distance expanded to perfectly match the substrate. For larger thickness, the lattice parameters were found to evolve towards those of bulk MgB_2_. All measurements, including ARPES, were performed *in situ*, under ultra-high vacuum conditions, to avoid contamination of the samples.

### Electronic structure and electron-phonon interaction

**Figure 2:**
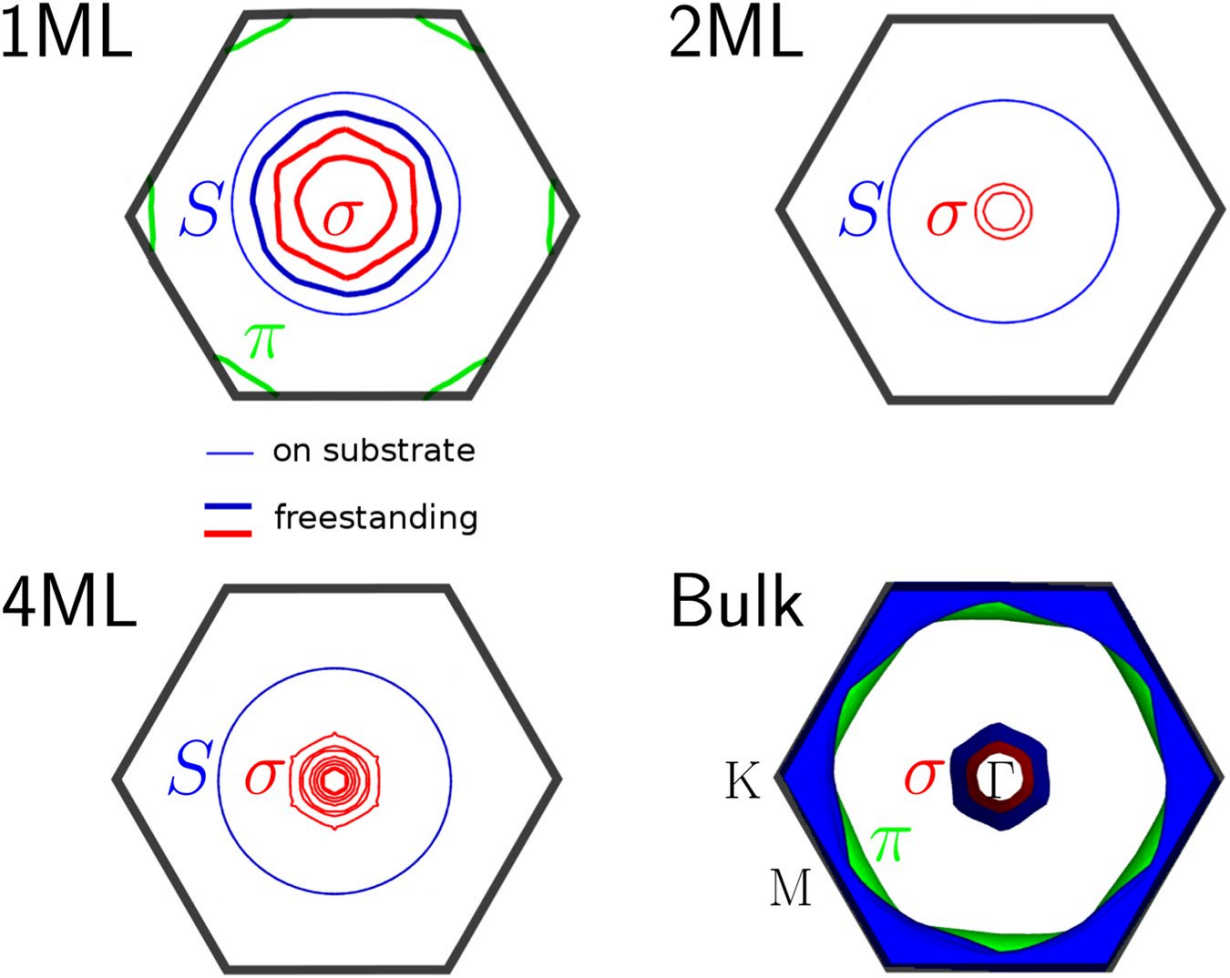
Fermi surface of ultrathin MgB_2_. The Fermi surface is depicted in the first hexagonal Brillouin zone (solid black line), as a function of the number of layers, for one monolayer (ML), two MLs, four MLs and bulk MgB_2_. For the few-monolayer structures, the Fermi surface effectively consists of contours. Only states localized in the layers close to the Mg-vacuum interface are depicted in this case. The bands are indicated by *σ*, *π* and $S$, the former two because of their similarity with bulk MgB_2_ and the latter to denote the surface character of the band. For one ML, the distinction in Fermi contours between the monolayer on the substrate (thin lines) and the freestanding monolayer (thick line) is significant, while for two and four MLs the *σ*-bands of both cases are indistinguishable. The positions of the high-symmetry **k**-points are indicated in the bottom right panel.

**Figure 3:**
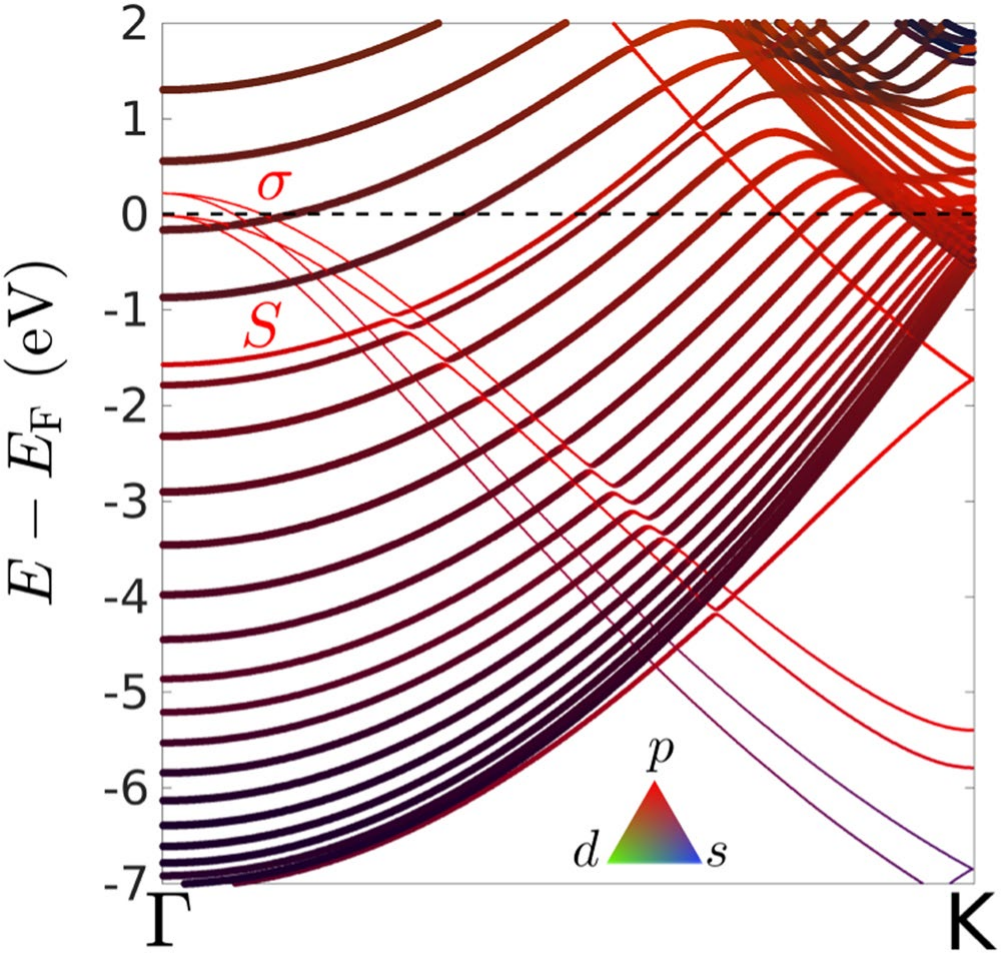
Calculated atomic character of the atomic orbitals of few-monolayer MgB_2_. Calculated band structure of two monolayers on the Mg-substrate, where colors denote the band character ($s$, $p$, $d$), as shown in the inset, and line thickness denotes the atomic species it originates from (thin for B and thick for Mg). Whereas in Fig. 2 only states localized in layers close to the Mg-vacuum interface were depicted, here all states are shown (including the states due to the Mg-substrate).

The electronic character of the bands is crucial in understanding the contribution of surface states in few-monolayer MgB_2_. The Fermi surface of MgB_2_, calculated using DFT as a function of the number of layers, is displayed in Fig. 2. The *σ*-band with ${p}_{x,y}$ character originates from covalent in-plane B-B bonds, while the *π*-band stems from out-of-plane B-B bonds with ${p}_{z}$ character. The Fermi level (${E}_{{\rm{F}}}$) is determined by the ionic character of the Mg-B bonds, through charge transfer. Using Bader charge analysis we found that in bulk MgB_2_ Mg-atoms donate approx. 0.8 electrons per B atom. In a freestanding ML, Mg donates 0.7 electrons to each B-atom, hence the similarity of its Fermi contour to the bulk Fermi surface. However, in the Fermi contour of few-monolayer MgB_2_, an additional spherical band is present. It is characteristic of the presence of the free Mg-surface, and therefore denoted $S$, for surface band. Originating directly from the Mg-atoms facing the vacuum, its band character consists predominantly (~90%) of Mg-$p$ states, as opposed to the B-$p$ character of the other bands. This Mg-$p$ character of band $S$ is shown in Fig. 3, where the orbitals calculated using DFT are projected onto the atomic orbitals.

**Figure 4:**
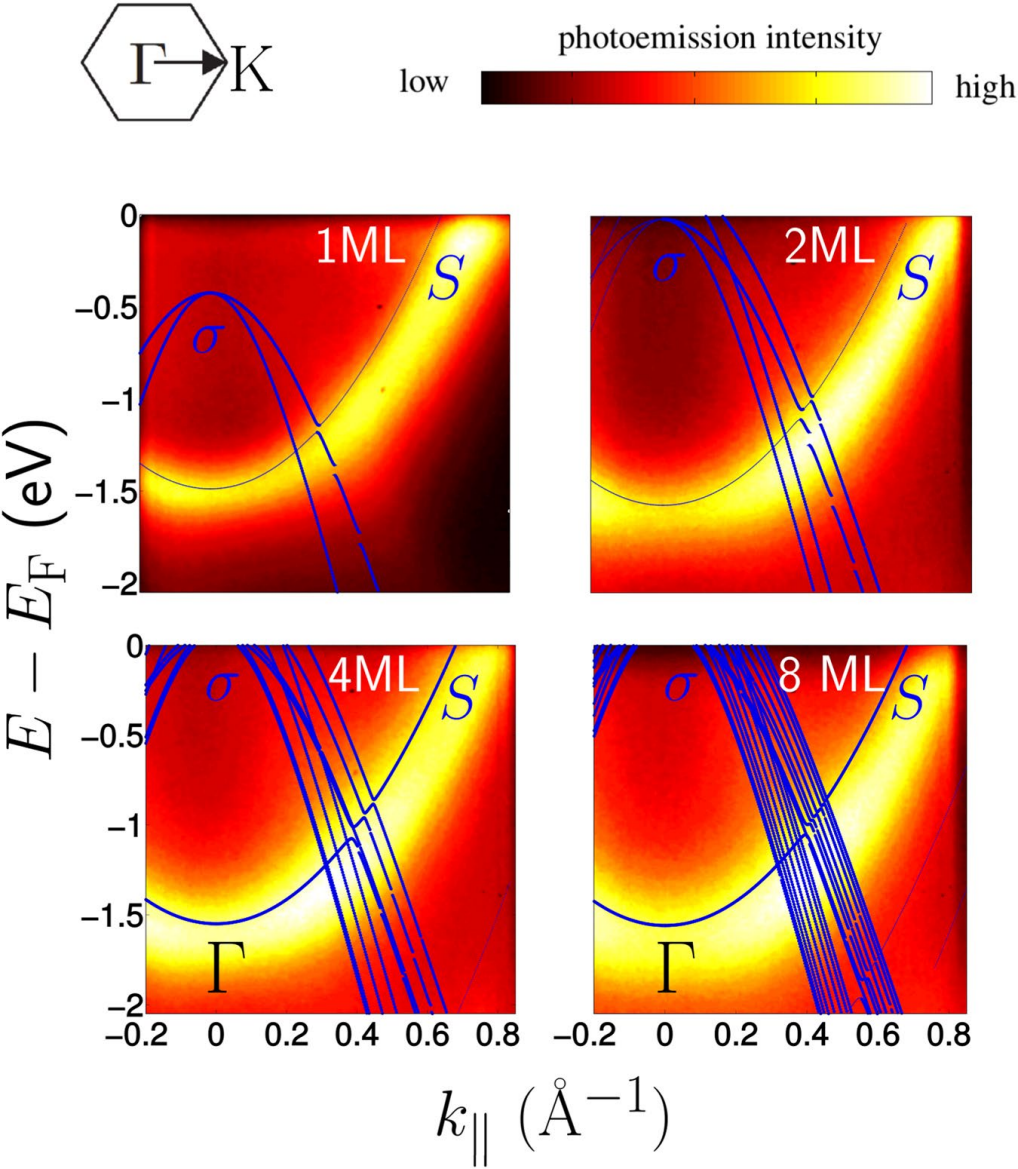
Room-temperature ARPES measurements of ultrathin MgB_2_. Data are shown for the valence bands of one, two, four, and eight MLs MgB_2_, along ${\rm{\Gamma }}$-K, with band structure calculations plotted on top. In the latter, thin lines indicate states originating for 75 to 90% from the layers adjacent to the Mg-vacuum interface, namely those states spatially localised within at most 2 nm from the top of the film – corresponding to the depth down to which ARPES can probe – while thick lines indicate states where this contribution exceeds 90%. The ARPES spectra were acquired using $h\nu =21.2$ eV incident radiation.

In one ML on the Mg-substrate, the single B-layer is sandwiched between two Mg-layers. As such, each atom in this B-layer receives 1.3 electrons from the two adjacent Mg-layers, in other words, it is *overdoped*. As a result, the *σ*-band is pushed down, as can be observed in Fig. 4 for 1 ML. The *σ* -band is thus eliminated as part of the Fermi surface of one ML of MgB_2_ on the Mg-substrate, as seen in Fig. 2. Band $S$, on the other hand, remains present as a very robust feature of ultrathin MgB_2_ films. For two MLs, the *σ* -band reappears at the Fermi level, and the difference in Fermi contours between freestanding and epitaxial structures vanishes. For four MLs and more, the *σ*-band starts converging towards its bulk limit. In contrast, the Mg-based surface band persists as a distinct and prominent feature. Owing to its ${p}_{z}$-character, the *π*-band of few-monolayer MgB_2_ hybridizes with Mg-${p}_{z}$ states of the substrate. This effect, due to Kronig-Penney-like interaction between Mg-layers in the substrate and the films, diminishes with an increasing number of layers. Therefore, hybridization with the substrate becomes weak in six MLs and more, and the *π*-band re-emerges at the Fermi level.

To verify the appearance of the surface band near ${E}_{{\rm{F}}}$ in the few-monolayer structures, we performed angle-resolved photoemission spectroscopy (ARPES) at room temperature, shown in Fig. 4 together with the calculated *σ*- and $S$- bands. The latter becomes increasingly localised near the surface for thicker films, explaining the increase in intensity of the ARPES signal. The ARPES signal from the *σ*-bands is significantly weaker than that of the $S$-band, especially in the one ML case where the *σ*-bands are not clearly visible in the ARPES data in Fig. 4 (but more clearly so at higher binding energies, such as in Fig. 1(b) of the Suppl. Mat.). The predicted depletion of the *σ*-bands for 1 ML MgB_2_ is nevertheless in line with the lack of intensity at ${\rm{\Gamma }}$ for MgB_2_ layers thicker than 1 ML, indicating that the *σ*-band is shifted towards lower binding energies in the 1 ML case, as pointed out by the calculations.

**Figure 5:**
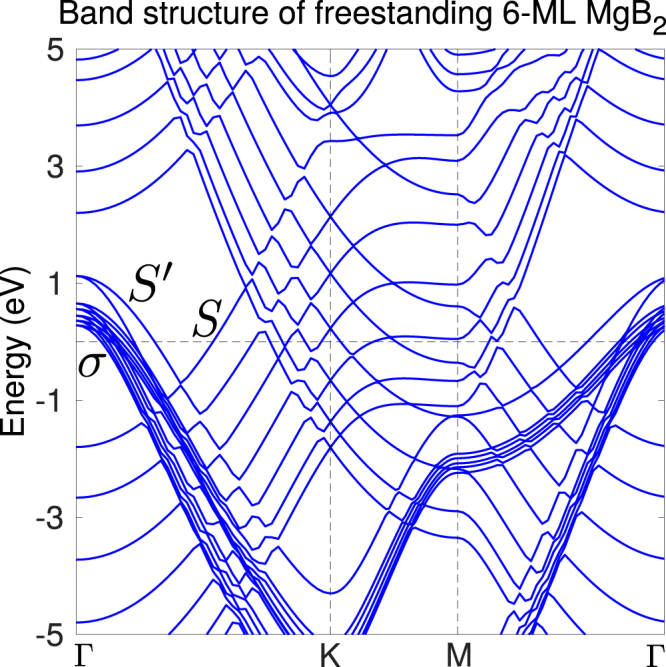
The calculated band structure of freestanding 6 ML thick MgB_2_. In addition to the surface band $S$, originating from the Mg-terminated site, the absence of the substrate opens an additional surface band, $S^{\prime}$, as a split-off band from the *σ*-bands. It arises from the B-layer facing vacuum instead of facing the substrate, so contrary to band $S$, it has the same B-$p$ character as the *σ*-bands.

Finally, there is an interesting difference between the electronic states in freestanding MgB_2_ films and those attached to the Mg(0001) substrate. We showed in Figs 2–4 that the *σ*-bands of films of 2 ML thickness and more, deposited on the Mg(001) substrate, lie close together. In freestanding form, on the contrary, one band splits off from the *σ*-bands, as shown in Fig. 5, for 6 ML thickness (treated further in the next section regarding its superconducting properties). This split-off band, band $S^{\prime}$, has B-${p}_{xy}$ character and originates from a surface state of the free B-surface, in the absence of the substrate.

In order to characterize the nucleation of superconductivity in few-monolayer MgB_2_ from electron-phonon interaction, we calculated the phonon spectrum and the electron-phonon coupling for few-monolayer MgB_2_ as well as bulk MgB_2_ from first-principles. Using density functional perturbation theory (DFPT), as implemented in ABINIT^53,54^, we determined the electron-phonon coupling coefficients ${g}_{{\bf{k}},{\bf{k}}+{\bf{q}}}^{\nu }$, where $\nu$ denotes the different phonon modes and ${\bf{k}}$ and ${\bf{q}}$ are wave vectors. The often used isotropic Eliashberg theory does not suffice to study multigap superconductivity and cannot explain the high transition temperatures of MgB_2_^41^. Therefore, we employed fully anisotropic Eliashberg theory with the ${g}_{{\bf{k}},{\bf{k}}{\boldsymbol{\text{+}}}{\bf{q}}}^{\nu }$ as input for the calculation of the superconducting properties, presented in the next section.

### Superconducting properties

**Figure 6:**
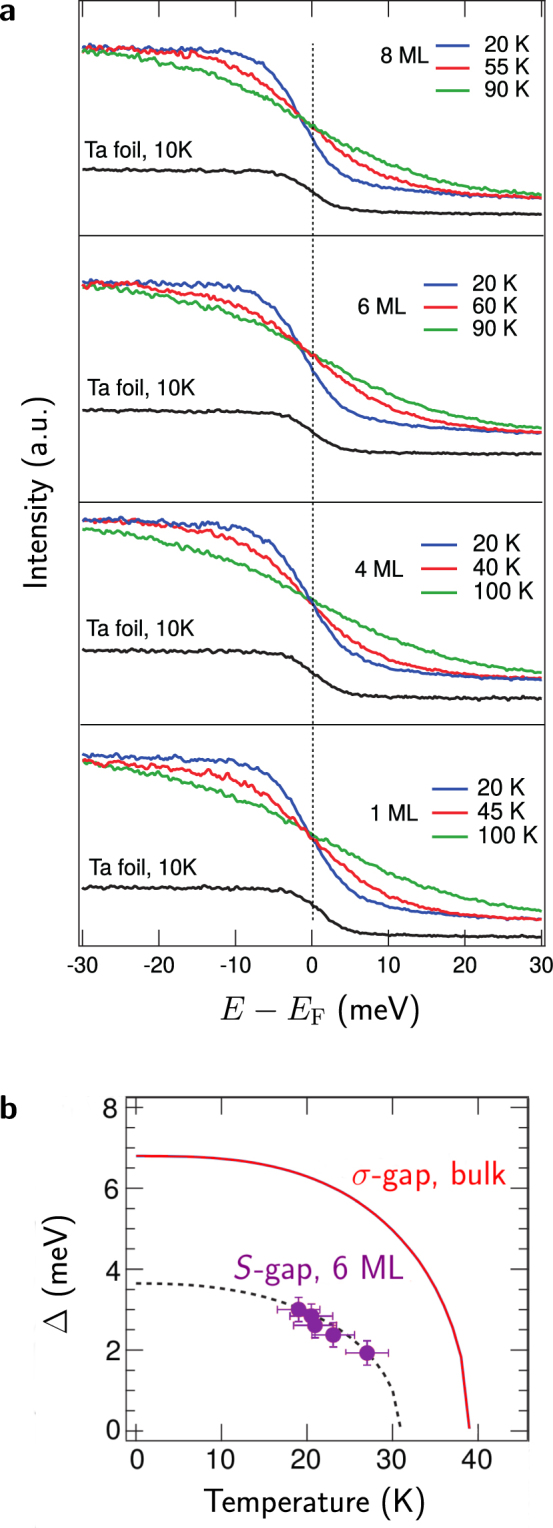
Low-temperature ARPES spectra and superconducting gap opening on the surface band. (**a**) ARPES spectra at low temperature (measured down to 20 K) of the Fermi level region of atomically thin MgB_2_ films, acquired using $h\nu =9.0$ eV incident radiation. The data were acquired at the k-points where the $S$ band crosses ${E}_{{\rm{F}}}$. One notices the superconducting gap for six and eight MLs at the lowest measured temperature, 20 K. The spectrum acquired on a Ta-foil is shown as a reference for the position of ${E}_{{\rm{F}}}$. (**b**) The evolution of the gap on the $S$-band with temperature, as measured using ARPES. A fit of the profile yields ${{\rm{\Delta }}}_{S}\mathrm{(0)}=3.6$ meV and ${T}_{{\rm{c}},S}\sim 31$ K, the critical temperature of superconductivity stemming from the surface band. The *σ*-gap profile of bulk MgB_2_ is added for comparison.

To study superconductivity stemming from the surface band in few-monolayer MgB_2_, we performed low-temperature ARPES measurements, down to 20 K. The results are shown in Fig. 6, together with the spectrum of the Fermi level region of the polycrystalline Ta foil, measured at 10 K, which was used as a reference for the position of ${E}_{{\rm{F}}}$. The spectra are acquired in the point where band $S$ crosses the Fermi level along the $\Gamma$-K direction. Given the very strong ARPES signal on the *S*-band (cf. Fig. 4), the superconducting gap opening on this band can be determined very accurately. Therefore, our experiment focusses on superconductivity in this band, unique to ultrathin MgB_2_. The position of the spectral edge in the valence band spectra of the one to four ML thick films remained the same at all temperatures and the saddle point along the slope coincided with the ${E}_{{\rm{F}}}$ position of the Ta reference. In contrast, a change in the position of the spectral edge with temperature is observed in Fig. 6a for the six and eight ML thick films. Moreover, this modification occurred only at low temperatures, as can be witnessed in the figure. Such shift towards higher binding energy of the spectral leading edge from the position of the reference ${E}_{{\rm{F}}}$ indicates a vanishing density of states at the Fermi level and therefore the opening of a superconducting gap^55^, ${\rm{\Delta }}$, in samples of six and eight ML thick. For samples thinner than six MLs no superconducting gap is observed in the temperature range attainable with our experimental set-up. Also, the proximity effect of the substrate is expected to play a role in suppressing superconductivity in the thinnest limit. Effectively, this comprises two kinds of proximity effects due to the substrate. Firstly, there is the purely electronic proximity effect for one ML MgB_2_, shown in Fig. 2, where charge transfer from the substrate to the film eliminates the *σ*-bands from the Fermi surface. The second kind of proximity effect stems from a transfer of Cooper pairs from the superconducting film to the metallic substrate, which also depletes superconductivity. Since purely from theory there is no reason why there would be no superconductivity in the thinner films^56^ it is very likely that this second kind of proximity effect inhibits superconductivity in films thinner than six MLs if grown on a metallic substrate.

For six MLs, we performed measurements at several temperatures in the range 20–30 K, shown in Fig. 6b. The gap width, ${\rm{\Delta }}$, was obtained by fitting the valence band spectra with a BCS spectral function multiplied by a Fermi-Dirac distribution and convoluted with the experimental Gaussian broadening, the width of which was determined from the Ta spectra. The measurement enables us to fit the superconducting gap as a function of temperature according to ${\rm{\Delta }}(T)={\rm{\Delta }}\mathrm{(0)}{(1-{(T/{T}_{{\rm{c}}})}^{p})}^{\mathrm{1/2}}$, from which we obtain $p=2.4$, ${\rm{\Delta }}\mathrm{(0)}=3.6$ meV, and the critical temperature of this gap, ${T}_{{\rm{c}},S}=31$ K.

This superconductivity originating from the surface state cannot be explained by previously proposed models of few-monolayer MgB_2_ based on the tight-binding formalism, in which surface states (electronic as well as vibrational) were entirely omitted^57^. So, to unravel the origin of the measured superconductivity in the surface band, and its relation to the other gaps, we employed fully anisotropic Eliashberg theory on a freestanding, six ML MgB_2_ film, using the electron-phonon coupling coefficients obtained from first-principles as input (more detailed information on which can be found in Methods). The calculated distribution of the gap opening specifically on band $S$ as a function of temperature is shown in Fig. 6a (dashed line). It is observed that this $S$-gap closes at ${T}_{{\rm{c}},S}=33$ K – in excellent agreement with the experimental value (${T}_{{\rm{c}},S}=31$ K). The same is true for the characteristic exponent $p$ of the temperature dependence of the gap opening on $S$, obtained as $p=2.2$ in the theory (vs. $p=2.4$ in the experiment). The calculated gap opening on band $S$, ${{\rm{\Delta }}}_{S}\mathrm{(0)}=3.3$ meV, is also in very good accordance with the ARPES result (${{\rm{\Delta }}}_{S}\mathrm{(0)}=3.6$ meV). In the anisotropic Eliashberg calculation we employed the standard value ${\mu }^{\ast }=0.13$ to model the electron-electron repulsion that counteracts Cooper pair formation^58^. From the close agreement between our theoretical predictions and experimental data it can be concluded that the screening modelled by ${\mu }^{\ast }$ does not change in the ultrathin limit in the case of MgB_2_, as opposed to, e.g., ultrathin TaS_2_ where it was proposed that a renormalization of this repulsion lies at the base of the increase in critical temperature in thinner samples^59^.

**Figure 7:**
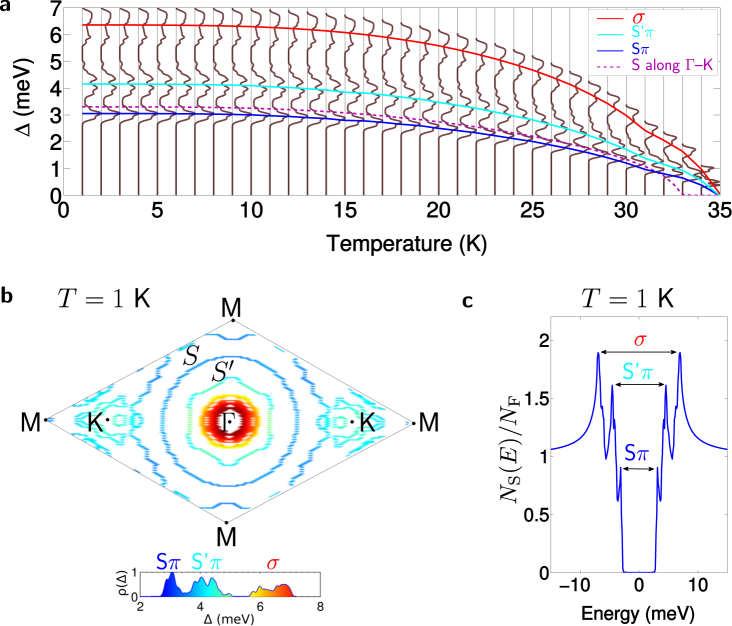
Superconducting gap spectrum from anisotropic Eliashberg theory calculations. (**a**) The computed temperature dependence of the superconducting gap spectrum, ${\rm{\Delta }}$, of six MLs of MgB_2_. The spectrum consists of three distinct contributions (domes), denoted $S\pi$, $S^{\prime} \pi$ and *σ* respectively, after the band contributions. The dome averages are also indicated, as well as the gap specifically opening on the $S$-band along $\Gamma -K$, for direct comparison to the experimental result in Fig. 5. The theoretical critical temperature ${T}_{{\rm{c}},S}=33$ K of this band and the strength of the gap are in excellent agreement with the experiment. (**b**) The calculated superconducting gap spectrum of six MLs of MgB_2_ at temperature $T=1$ K, plotted on the Fermi surface. Inset: The distribution of the gap, $\rho ({\rm{\Delta }})$, whereby the three distinct domes are indicated, showing their origin from the bands of the Fermi surface. (**c**) The density of states in the superconducting state at 1 K, with three distinct peaks related to the three domes in the superconducting gap spectrum.

The gap spectrum displayed in Fig. 7a is very rich, and the $S$-gap is certainly not the only contribution. In order to understand the physical origin of this rich spectrum, we plot the calculated distribution of the gap on the Fermi surface, $\rho ({\rm{\Delta }})$, at 1 K, in Fig. 7b. It consists of three domes of which two (with values ~3–3.5 meV and ~6–7 meV) are comparable to the *π*- and *σ*-gaps of bulk MgB_2_ respectively. However, the free surfaces provide additional contributions that phonons couple to, akin to, e.g., the interlayer state crucial for superconductivity in functionalized graphene^17,19,20^. The Mg-based band $S$, resulting from the Mg-vacuum interface, contributes to the first dome by hybridizing with the *π*-gap (denoted $S\pi$-dome). The gap on band $S^{\prime}$ is in the range 4–5 meV and is also mixed with the *π*-gap, forming the central dome of $\rho ({\rm{\Delta }})$ denoted $S^{\prime} \pi$. In the experiment, this B-surface is bound to the substrate, and as a result band $S^{\prime}$ approaches the *σ*-bands.

As bands $S$ and $S^{\prime}$ stem from the free surfaces of opposite sides of the film, what happens to band $S^{\prime}$ does not influence the gap opening on the $S$-band of the Mg-surface. Therefore, separate control of the contributions of these two surface states to the gap spectrum can be achieved by means of chemical functionalization of one or either of these free surfaces. Specifically, we envisage that the surface bands can be eliminated from the Fermi surface by means of adatoms on the Mg-side (for the $S$-band) and/or the B-side (for the $S^{\prime}$-band). With elements with a low atomic number, such as hydrogen, the charge transfer between the adatoms and the film can be limited, such that the *σ*- and *π*-states would be largely unaltered under the influence of this functionalization. This provides for a unique (possibly local) control of the superconducting gap spectrum through easily accessible surfaces.

Furthermore, the different domes in the superconducting gap spectrum with their distinct contributions in six MLs MgB_2_, $S\pi$, $S^{\prime} \pi$ and *σ*, are robust under the influence of temperature, persisting even at temperatures close to the overall critical temperature of six MLs of MgB_2_, ${T}_{{\rm{c}}}=35$ K. This is concluded from the temperature dependence of the full gap distribution, $\rho ({\rm{\Delta }})$, displayed in Fig. 7a, together with the weighted averages of the different domes. The effect of the surface contributions on the density of states (DOS) in the superconducting state, ${N}_{{\rm{S}}}$, calculated from the superconducting gap spectrum, is also very pronounced, as displayed in Fig. 7c. ${N}_{{\rm{S}}}$ exhibits three sharply resolved peaks, corresponding to the three domes in $\rho ({\rm{\Delta }})$, whereas in bulk MgB_2_ the DOS in the superconducting state consists of only two peaks, corresponding to the *σ*- and *π*-gaps (as shown both in theory^42^ and in scanning-tunneling microscopy experiments^60^). As such, the tunneling properties undergo equally radical change going from bulk to ultrathin MgB_2_.

## Discussion

Our combined theoretical and experimental study of few-monolayer MgB_2_ unambiguously reveals that surface states drastically change superconductivity in atomically thin superconductors. The surface states in question stem from the outer layers of the material itself, i.e., the free surfaces facing vacuum, without need for any specific adatoms, intercalants or substrates that were previously shown to stimulate superconductivity in the two-dimensional limit. Moreover, the impact of free surfaces on superconductivity is inherent to atomically thin materials, but still susceptible to further chemical functionalization and nanoscale engineering. Our measured and calculated enriched gap spectrum of six MLs of MgB_2_ proves that these surface states can be exploited to qualitatively change the multigap nature of superconductivity in the atomically thin limit. We showed furthermore that the condensate originating from the surface state hybridizes with the *π*-condensate in few-monolayer MgB_2_. This is completely different from bulk-sized MgB_2_ where ARPES experiments indicate near-degeneracy of a surface state gap with the *σ*-gap, without any clear influence on the measured two-gap superconductivity^39^. This comparison implies a strong evolution of the superconducting gap spectrum, and strong changes in the interplay and hybridization between condensates when thinning MgB_2_ layer by layer. Therefore, as an outlook of this work, measurements below 20 K are foreseen to explore possible superconductivity in the thinnest structures of MgB_2_ – in line with theoretical predictions presented in ref.^56^. In that limit, our results indicate a considerable role of the substrate since, e.g., the Mg-substrate eliminates several electronic bands in case of a single ML of MgB_2_ (cf. Fig. 2a). Non-metallic substrates would exclude the possibility of Cooper-pairs escaping and would thus make the superconducting state even more robust. On a related note, recent experimental advances enable non-epitaxial fabrication of not only weakly bound layered materials, e.g., NbSe_2_^14^, but also of ultrathin compounds with ionic interlayer bonds, like MgB_2_^61^. It is thus envisioned that the full potential of superconductivity from free surfaces can be explored using the available technology and can be further incorporated in, e.g., atomically thin devices aimed at cryogenic computing.

## Methods

### Sample Preparation and characterization

The Mg(0001) substrate was prepared using cycles of sputtering and annealing at 493 K. During molecular beam epitaxy (MBE) of Mg and B from pure sources, with atomic flux ratio Mg:B = 3:2, the pressure was kept below ${10}^{-9}$ mbar, while the substrate was held at 475 ± 15 K. For the thinnest films (<4 ML), deposition of B was sufficient, whereas for thicker layers co-deposition of Mg and B was needed. We monitored the film thickness through the evaporation rate, using the calibrated attenuation of the substrate core level photoemission peaks when evaporating B on Mg(0001) and Mg on a copper plate. Surface X-ray diffraction (SXRD) was carried out at the ALOISA beamline of the Elettra Synchrotron, Trieste, Italy^62^.

### Angle Resolved Photoemission Spectroscopy

Angle-resolved photoelectron spectroscopy (ARPES) was performed at the BaDElPh beamline of the Elettra Synchrotron^63^ to map the variations in the electronic band structure of MgB_2_ as a function of film thickness. The spectra were collected both at room temperature and at low temperatures with a SPECS Phoibos-150 hemispherical electron analyzer, equipped with a 2D CCD sensor. The nominal energy resolution of the detector was 5 meV and its nominal angular resolution was 0.15°. In the low-temperature ARPES experiments, the samples were cooled using a liquid He-cryostat, and the temperature was measured by means of silicon diodes. The energy resolution and stability of the photon beam energy throughout the course of the experiment was monitored using photoemission spectra around the Fermi level of the clean Mg(0001) at the $\Gamma$ point of the Brillouin zone and that of a polycrystalline Ta-foil, in electric contact with the sample. More detailed information on stability and resolution in the ARPES measurements can be found in Supplementary Information, section 1.

### Density Functional (Perturbation) Theory

Our density functional theory (DFT) calculations make use of the Perdew-Burke-Ernzerhof (PBE) functional implemented within a planewave basis in the VASP code. Electron-ion interactions are treated using projector augmented wave (PAW) potentials, taking into account Mg-2*p* ^6^3*s* ^2^ and B-2*s* ^2^2*p* ^1^ as valence electrons. An energy cutoff of 450 eV for the planewave basis was used, to achieve convergence of the total energy below 1 meV per atom. To obtain a very accurate description of the Fermi surface, a very dense $35\times 35\times 31$ $\Gamma$-centered Monkhorst-Pack **k**-point grid is used for bulk MgB_2_, and a $35\times 35\times 1$ grid for few-monolayer structures. The lattice parameters were obtained using a conjugate-gradient algorithm so that forces on each atom were minimized below 1 meV/Å. Subsequently, density functional perturbation theory (DFPT) calculations were carried out within the framework of ABINIT, keeping the same valence electrons, and also using the PBE functional. The crystal structure was optimized again in ABINIT, with no significant differences with the VASP results. The total number of perturbations due to atomic displacements to be treated (in other words, the number of phonon branches) amounts to $3\cdot {N}_{{\rm{atoms}}}=54$ for six MLs. In this way, the phonon spectrum and electron-phonon coupling coefficients, matrix elements of the perturbative part of the Hamiltonian^54^ are obtained. We carried out the DFPT calculations on a $22\times 22\times 1$ electronic k-point grid and a $11\times 11\times \mathrm{1\ }$ q-point grid, a subgrid of the k-point grid, for the phonons. The DFPT calculations are performed on freestanding six MLs, thus the interface state between film and substrate, as well as interface phonons are deliberately omitted. The phonons and electron-phonon coupling of six ML and bulk MgB_2_ are discussed in Supplementary Information, section 2.

### Anisotropic Eliashberg Theory

The anisotropic Eliashberg equations were solved self-consistently in Matsubara space using the Uppsala superconductivity (UppSC) code, starting from the electron and phonon band structures and electron-phonon coupling obtained with DFPT, after which the converged solutions were analytically continued to real frequencies (see ref.^42^ for further details). In this scheme, we iterated until convergence better than 10^−6^ on the relative gap values was reached. In all calculations, we employed standard ${\mu }^{\ast }=0.13$ for the Coulomb repulsion between electrons in the Cooper pairs, and cutoff-frequency of 0.55 eV.

### Data availability statement

The datasets generated during and/or analysed during the current study are available from the corresponding author on reasonable request.

## Electronic supplementary material

Supplementary Information

## References

[1] Saito Y, Nojima T, Iwasa Y (2016). Highly crystalline 2D superconductors. Nat. Rev. Mater..

[2] Uchihashi T (2017). Two-dimensional superconductors with atomic-scale thickness. Supercond. Sci. Technol..

[3] Ozer MM, Thompson JR, Weitering HH (2006). Hard superconductivity of a soft metal in the quantum regime. Nat. Phys..

[4] Qin S, Kim J, Niu Q, Shih C (2009). Superconductivity at the two-dimensional limit. Science.

[5] Zhang T (2010). Superconductivity in one-atomic-layer metal films grown on Si(111). Nat. Phys..

[6] Uchihashi T, Mishra P, Aono M, Nakayama T (2011). Macroscopic superconducting current through a silicon surface reconstruction with indium adatoms: Si(111)−(7^1/2^×3^1/2^)-In. Phys. Rev. Lett..

[7] Yamada M, Hirahara T, Hasegawa S (2013). Magnetoresistance measurements of a superconducting surface state of In-induced and Pb-induced structures on Si(111). Phys. Rev. Lett..

[8] Brun C (2014). Remarkable effects of disorder on superconductivity of single atomic layers of lead on silicon. Nat. Phys..

[9] Yoshizawa S (2014). Imaging Josephson vortices on the surface superconductor Si(111)−(7^1/2^×3^1/2^)-In using a scanning tunneling microscope. Phys. Rev. Lett..

[10] Matetskiy AV (2015). Two-dimensional superconductor with a giant Rashba effect: One-atom-layer Tl-Pb compound on Si(111). Phys. Rev. Lett..

[11] Kim H (2016). Electrical conductivity through a single atomic step measured with the proximity-induced superconducting pair correlation. Phys. Rev. Lett..

[12] Zhang H-M (2015). Detection of a superconducting phase in a two-atom layer of hexagonal Ga film grown on semiconducting GaN(0001). Phys. Rev. Lett..

[13] Zhang YJ, Yoshida M, Suzuki R, Iwasa Y (2015). 2D crystals of transition metal dichalcogenide and their iontronic functionalities. 2D Mater..

[14] Cao Y (2015). Quality heterostructures from two-dimensional crystals unstable in air by their assembly in inert atmosphere. Nano Lett..

[15] Ugeda MM (2016). Characterization of collective ground states in single-layer NbSe_2_. Nat. Phys..

[16] Xi X (2016). Ising pairing in superconducting NbSe_2_ atomic layers. Nat. Phys..

[17] Profeta G, Calandra M, Mauri F (2012). Phonon-mediated superconductivity in graphene by lithium deposition. Nat. Phys..

[18] Guzman DM, Alyahyaei HM, Jishi RA (2014). Superconductivity in graphene-lithium. 2D Mater..

[19] Ludbrook BM (2015). Evidence for superconductivity in Li-decorated monolayer graphene. Proc. Natl. Acad. Sci..

[20] Kanetani K (2012). Ca intercalated bilayer graphene as a thinnest limit of superconducting C_6_Ca. Proc. Natl. Acad. Sci..

[21] Chapman J (2016). Superconductivity in Ca-doped graphene laminates. Sci. Rep..

[22] Verbitskiy NI (2016). Environmental control of electronphonon coupling in barium doped graphene. 2D Mater..

[23] Geng D (2017). Controlled growth of ultrathin Mo_2_C superconducting crystals on liquid Cu surface. 2D Mater..

[24] Bollinger AT (2011). Superconductor-insulator transition in La_2−*x*_Sr_*x*_Cuo_4_ at the pair quantum resistance. Nature.

[25] Ge J (2015). Superconductivity above 100 K in single-layer FeSe films on doped SrTiO_3_. Nat. Mater..

[26] Wang Q (2015). Thickness dependence of superconductivity and superconductorinsulator transition in ultrathin FeSe films on SrTiO_3_ (001) substrate. 2D Mater..

[27] Zhao W, Chang C-Z, Xi X, Mak KF, Moodera JS (2016). Vortex phase transitions in monolayer FeSe film on SrTiO_3_. 2D Mater..

[28] Li F (2016). Atomically resolved FeSe/SrTiO_3_ (001) interface structure by scanning transmission electron microscopy. 2D Mater..

[29] Huang ZC (2016). Electronic structure and superconductivity of single-layer FeSe on Nb:SrTiO_3_/LaAlO_3_ with varied tensile strain. 2D Mater..

[30] Zhang J-J, Dong S (2016). Prediction of above 20 K superconductivity of blue phosphorus bilayer with metal intercalations. 2D Mater..

[31] Golod, T., Iovan, A. & Krasnov, V. M. Single Abrikosov vortices as quantized information bits. *Nat. Commun*. **6** (2015). 10.1038/ncomms9628 PMC4633956 26456592

[32] Najafi F (2015). On-chip detection of non-classical light by scalable integration of single-photon detectors. Nat. Commun..

[33] Lowell PJ (2016). A thin-film cryotron suitable for use as an ultra-low-temperature switch. Appl. Phys. Lett..

[34] Geim AK, Grigorieva IV (2013). Van der Waals heterostructures. Nature.

[35] Saito Y (2016). Superconductivity protected by spin-valley locking in ion-gated MoS_2_. Nat. Phys..

[36] Kortus J, Mazin II, Belashchenko KD, Antropov VP, Boyer LL (2001). Superconductivity of metallic boron in MgB_2_. Phys. Rev. Lett..

[37] Liu AY, Mazin II, Kortus J (2001). Beyond Eliashberg superconductivity in MgB_2_: Anharmonicity, two-phonon scattering, and multiple gaps. Phys. Rev. Lett..

[38] Golubov AA (2002). Specific heat of MgB_2_ in a one- and a two-band model from first-principles calculations. J. Phys. Condens. Matter.

[39] Souma S (2003). The origin of multiple superconducting gaps in MgB_2_. Nature.

[40] Margine ER, Giustino F (2013). Anisotropic Migdal-Eliashberg theory using Wannier functions. Phys. Rev. B.

[41] Choi HJ, Roundy D, Sun H, Cohen ML, Louie SG (2002). The origin of the anomalous superconducting properties of MgB_2_. Nature.

[42] Aperis A, Maldonado P, Oppeneer PM (2015). *Ab initio* theory of magnetic-field-induced odd-frequency two-band superconductivity in MgB_2_. Phys. Rev. B.

[43] Milošević MV, Perali A (2015). Emergent phenomena in multicomponent superconductivity: an introduction to the focus issue. Supercond. Sci. Technol..

[44] Stanev V, Tešanović Z (2010). Three-band superconductivity and the order parameter that breaks time-reversal symmetry. Phys. Rev. B.

[45] Garaud J, Carlström J, Babaev E (2011). Topological solitons in three-band superconductors with broken time reversal symmetry. Phys. Rev. Lett..

[46] Komendová L, Chen Y, Shanenko AA, Milošević MV, Peeters FM (2012). Two-band superconductors: Hidden criticality deep in the superconducting state. Phys. Rev. Lett..

[47] Bekaert J (2016). Anisotropic type-I superconductivity and anomalous superfluid density in OsB_2_. Phys. Rev. B.

[48] Talantsev EF (2017). On the origin of critical temperature enhancement in atomically thin superconductors. 2D Mater..

[49] Hahn, T. (ed.) International Tables for Crystallography, Vol. *A* (Springer, 2005).

[50] Kresse G, Furthmüller J (1996). Efficient iterative schemes for ab initio total-energy calculations using a plane-wave basis set. Comp. Mater. Sci..

[51] Cepek C (2004). Epitaxial growth of MgB_2_(0001) thin films on magnesium single-crystals. Appl. Phys. Lett..

[52] Petaccia L (2006). Characterization of high-quality MgB_2_(0001) epitaxial films on Mg(0001). New J. Phys..

[53] Gonze X (2009). Abinit: First-principles approach to material and nanosystem properties. Comput. Phys. Commun..

[54] Savrasov SY, Savrasov DY (1996). Electron-phonon interactions and related physical properties of metals from linear-response theory. Phys. Rev. B.

[55] Harris JM (1997). Measurement of an anisotropic energy gap in single plane Bi_2_Sr_2−*x*_ La_*x*_CuO_6+ *δ*_. Phys. Rev. Lett..

[56] Bekaert J, Aperis A, Partoens B, Oppeneer PM, Milošević M (2017). Evolution of multigap superconductivity in the atomically thin limit: Strain-enhanced three-gap superconductivity in monolayer MgB_2_. Phys. Rev. B.

[57] Szałowski K (2006). Critical temperature of MgB_2_ ultrathin superconducting films: BCS model calculations in the tight-binding approximation. Phys. Rev. B.

[58] Grimvall, G. *The electron-phonon interaction* (North Holland Publishing Co., 1981).

[59] Navarro-Moratalla E (2016). Enhanced superconductivity in atomically thin TaS_2_. Nat. Commun..

[60] Chen K (2012). Momentum-dependent multiple gaps in magnesium diboride probed by electron tunnelling spectroscopy. Nat. Commun..

[61] Das SK, Bedar A, Kannan A, Jasuja K (2015). Aqueous dispersions of few-layer-thick chemically modified magnesium diboride nanosheets by ultrasonication assisted exfoliation. Sci. Rep..

[62] Floreano L (1999). Performance of the grating-crystal monochromator of the ALOISA beamline at the elettra synchrotron. Rev. Sci. Instrum..

[63] Petaccia L (2009). BaD ElPh: A 4m normal-incidence monochromator beamline at Elettra. Nucl. Instr. Meth. Phys. Res. Section A: Accelerators, Spectrometers, Detectors and Associated Equipment.

