# Supplementary material for: Free surfaces recast superconductivity in few-monolayer MgB2: Combined first-principles and ARPES demonstration
Source: Sci Rep. 2017 Oct 31;7:14458. doi: 10.1038/s41598-017-13913-z (PMC5663715; doi:10.1038/s41598-017-13913-z)
Supplement: Supplementary file 1 — Supplementary Information [file 41598_2017_13913_MOESM1_ESM.pdf]

Supplementary Information on "Free surfaces recast superconductivity in few-monolayer MgB_2: Combined first-principles and ARPES demonstration"

J. Bekaert[1*], L. Bignardi[2,3], A. Aperis[4], P. van Abswoude[2], C. Mattevi[5,6], S. Gorovikov[7,8], L. Petaccia[7], A. Goldoni[7], B. Partoens[1], P. M. Oppeneer[4], F. M. Peeters[1], M. V. Milošević[1*], P. Rudolf[2*], C. Cepek[5]

September 25, 2017

1. Condensed Matter Theory group, Department of Physics, University of Antwerp, Groenenborgerlaan 171, B-2020 Antwerp, Belgium.

2. Zernike Institute for Advanced Materials, University of Groningen, Nijenborgh 4, 9747AG Groningen, The Netherlands.

3. Current address: Elettra Sincrotrone Trieste, Strada Statale 14 km.163.5, I-34149 Trieste, Italy.

4. Department of Physics and Astronomy, Uppsala University, Box 516, SE-751 20 Uppsala, Sweden.

5. IOM-CNR, Laboratorio TASC, Strada Statale 14 km.163.5, I-34149 Trieste, Italy.

6. Current address: Department of Materials, Imperial College London, Exhibition road, SW7 2AZ, London, United Kingdom.

7. Elettra Sincrotrone Trieste, Strada Statale 14 km.163.5, I-34149 Trieste, Italy.

8. Current address: Canadian Light Source Inc., 44 Innovation Blvd., Saskatoon, SK S7N 2V3, Canada.

* Correspondence to J.B. (email: jonas.bekaert@uantwerpen.be) and M.V.M. (email: milorad.milosevic@uantwerpen.be) for queries about theory, and to P.R. (email: p.rudolf@rug.nl) for queries about the experiment and for sample requests.

1 Stability and resolution in ARPES

A precise knowledge of the energy resolution and stability of the photon beam energy throughout the course of experiment is of capital importance to be able to determine the opening of a superconducting gap of a few meV [1]. Therefore we measured photoemission spectra of the Fermi level of the clean Mg(0001) at the Γ-point of the Brillouin zone and that of a polycrystalline Ta foil, fastened on the back of the cryostat, in electric contact with the sample. The sample was cooled by means of a liquid He-cryostat. The temperature was measured with silicon diodes and the minimum value reached on the Ta-foil, in direct contact with the cryostat, was 9 K, while on the sample it amounted to 19 K.

The upper panel of Fig. 1 shows photoemission spectra of the Fermi level region acquired with a photon energy of 9 eV on Ta foil at different temperatures, ranging from 9 to 105 K. The spectra were acquired using the same experimental conditions (spectrometer and beamline settings, photon energy, sample temperatures) and on the same experimental time scale employed during the measurements of the MgB_2 films (several hours, necessary to cool the sample from room temperature to low temperature).

Consequently, the Fermi level position returns a direct measure of the stability of the photon energy and of the experimental apparatus, which could be critical when long acquisition times are required to measure a very small energy gap. The spectra were fitted with a Fermi-Dirac function, multiplied by a constant density of states function and convoluted with a Gaussian broadening function, whose full width at half maximum (FWHM) was extracted as a parameter of the fit and taken as the spectral broadening due to the spectrometer (energy resolution of the spectra acquired on a short-time scale, i.e. a few minutes). The beamline energy stability extracted by using several measurements at different temperatures was ca. 0.17 ± 0.20 meV (due to small variations in photon energy and in the power supplies), as reported in the top inset panel, whilst the average FWHM of the Gaussian broadening of each spectrum was 5.9 ± 0.2 meV. This ensures a good energy resolution.
As another reference, spectra of the clean Mg(0001) surface were acquired together with the spectrum of the Fermi level region of the Ta foil, as reported in the lower panel of Fig 1. For this measurement the energy stability was ca. 0.25 meV, in good agreement with the energy stability of the Ta foil. In this case, the measured Fermi level E_{F} of the Mg(0001) crystal fluctuated within 0.25 meV, due to small changes in photon energy, similar to the values found for the measurements on the Ta foil.

Room-temperature angle-resolved photoemission experiments were presented in the main manuscript. Here, we supplement this by Fig. 1b, over a broader energy range, as well as the corresponding calculated bands. Extra features that can be observed in this broader range

include the π-band around $k_{\parallel} = 0.8$ Å^{-1} in the energy range -5 to -4.5 eV below E_F.

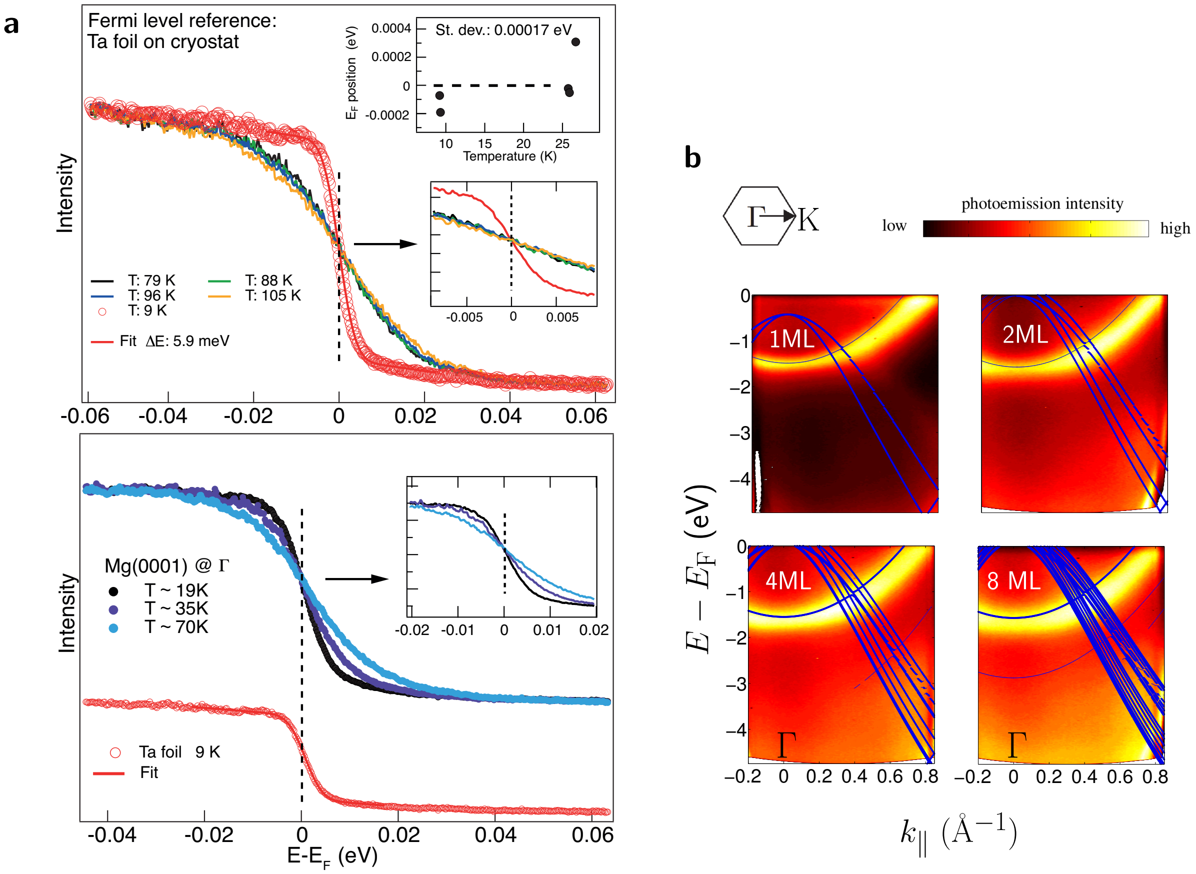

Figure 1: Reference photoemission spectra and extended ARPES measurements. (a) Photoemission spectra of the Fermi level region of a Ta-foil (top panel) and of the clean Mg(0001) substrate (bottom panel), acquired at normal emission with a photon energy of 9 eV. (b) Extended room-temperature ARPES measurements for several film thicknesses, with theoretical band structures plotted on top.

2 Phonons and electron-phonon coupling in six monolayers and bulk MgB_2

The results of the DFPT calculations for six MLs of MgB_2 and for the bulk, for comparison, are displayed in Fig. 2. One observes that the phonon band structures and phonon density of states of the six MLs structure show traits of their bulk counterparts. In both cases there is a clear separation between B- and Mg-related modes, where the latter constitute the lower-energy modes due to the higher atomic mass of Mg. The main difference between both is an extra contribution in the six ML case near 20 meV due to the lower acoustic mode along the perpendicular direction (the so-called ZA-mode). We calculated the isotropic Eliashberg

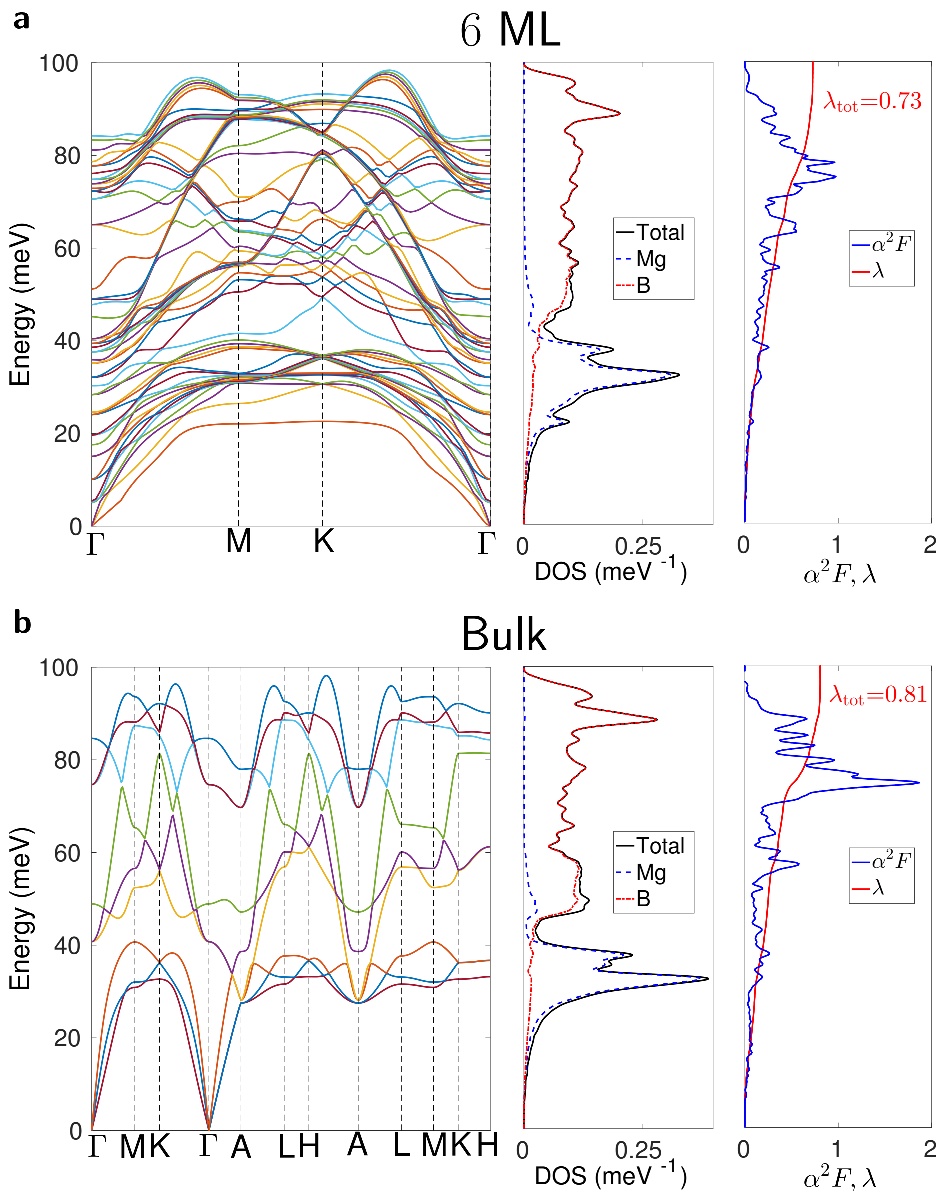

Figure 2: Calculated phonon dispersion and electron-phonon coupling of few-layer MgB_2. Phonon band structure, phonon density of states (DOS) and the isotropic Eliashberg function α^2F and isotropic electron-phonon coupling λ calculated using density-functional perturbation theory for (a) 6 monolayer thick MgB_2 and (b) bulk MgB_2.

functions through [2]

$$\alpha^2F(\omega) = \sum_{\mathbf{kq}} \alpha^2F(\mathbf{k}, \mathbf{k}+\mathbf{q}, \omega) = N(E_\mathrm{F}) \sum_{\mathbf{kq}\nu} |g^{\nu}_{\mathbf{k},\mathbf{k}+\mathbf{q}}|^2 \delta(\omega - \omega_{\mathbf{q}\nu}) \ . \tag{1}$$

The results in Fig. 2 show that the strongest electron-phonon interaction resides for both 6 MLs and bulk in the B-related optical modes around 70 meV. The eigen displacements of

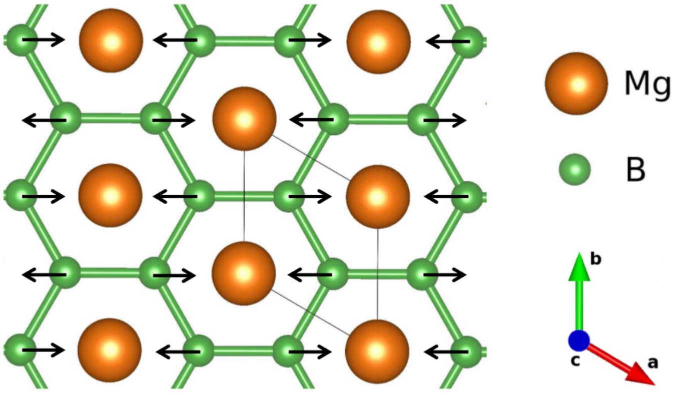

Figure 3: The E_{2g} phonon mode of the B-atoms that gives the strongest contribution to the electron-phonon coupling.

this mode, the E_2g mode, are depicted in Fig.3. The total electron-phonon coupling constant

$$\lambda_{\rm tot} = 2\int_0^\infty {\rm d}\omega\omega^{-1}\alpha^2 F(\omega)\ . \tag{2}$$

amounts to $\lambda_{\rm tot} = 0.81$ for bulk MgB_2 compared to $\lambda_{\rm tot} = 0.73$ for six MLs if evaluated isotropically.

In our anisotropic Eliashberg calculations we use the extended Eliashberg function $\alpha^2 F(\mathbf{k}, \mathbf{k}+\mathbf{q}, \omega)$, obtained by removing the summations over $\mathbf{k}$ and $\mathbf{q}$ in Eq. 1, to solve the Eliashberg equations for the gap spectrum numerically.

References

[1] Petaccia, L. *et al.* BaD ElPh: A 4m normal-incidence monochromator beamline at Elettra. *Nucl. Instr. Meth. Phys. Res. Section A: Accelerators, Spectrometers, Detectors and Associated Equipment* **606**, 780-784 (2009).

[2] Grimvall, G. *The electron-phonon interaction* (North Holland Publishing Co., 1981).
